# The Value of Whole-Genome Sequencing for Mitochondrial DNA Population Studies: Strategies and Criteria for Extracting High-Quality Mitogenome Haplotypes

**DOI:** 10.3390/ijms23042244

**Published:** 2022-02-17

**Authors:** Kimberly Sturk-Andreaggi, Joseph D. Ring, Adam Ameur, Ulf Gyllensten, Martin Bodner, Walther Parson, Charla Marshall, Marie Allen

**Affiliations:** 1 Department of Immunology Genetics and Pathology, Uppsala University, Uppsala 751 08, Sweden; adam.ameur@igp.uu.se (A.A.); ulf.gyllensten@igp.uu.se (U.G.); 2 Armed Forces Medical Examiner System’s Armed Forces DNA Identification Laboratory (AFMES-AFDIL), Dover Air Force Base, Dover, DE 19902, USA; joseph.d.ring2.ctr@mail.mil (J.D.R.); charla.k.marshall.ctr@mail.mil (C.M.); 3 SNA International, LLC, Alexandria, VA 22314, USA; 4 Institute of Legal Medicine, Medical University of Innsbruck, Innsbruck 6020, Austria; martin.bodner@i-med.ac.at (M.B.); walther.parson@i-med.ac.at (W.P.); 5 Forensic Science Program, The Pennsylvania State University, University Park, PA 16801, USA

**Keywords:** whole-genome sequencing, next-generation sequencing, massively parallel sequencing, mitochondrial DNA, nuclear elements of mtDNA, NUMTs, heteroplasmy

## Abstract

Whole-genome sequencing (WGS) data present a readily available resource for mitochondrial genome (mitogenome) haplotypes that can be utilized for genetics research including population studies. However, the reconstruction of the mitogenome is complicated by nuclear mitochondrial DNA (mtDNA) segments (NUMTs) that co-align with the mtDNA sequences and mimic authentic heteroplasmy. Two minimum variant detection thresholds, 5% and 10%, were assessed for the ability to produce authentic mitogenome haplotypes from a previously generated WGS dataset. Variants associated with NUMTs were detected in the mtDNA alignments for 91 of 917 (~8%) Swedish samples when the 5% frequency threshold was applied. The 413 observed NUMT variants were predominantly detected in two regions (nps 12,612–13,105 and 16,390–16,527), which were consistent with previously documented NUMTs. The number of NUMT variants was reduced by ~97% (400) using a 10% frequency threshold. Furthermore, the 5% frequency data were inconsistent with a platinum-quality mitogenome dataset with respect to observed heteroplasmy. These analyses illustrate that a 10% variant detection threshold may be necessary to ensure the generation of reliable mitogenome haplotypes from WGS data resources.

## 1. Introduction

Whole-genome sequencing (WGS) data generated for genetic variation studies are often submitted to public databases, such as GenBank [1], or made publicly available as part of the publication. Consequently, public WGS datasets provide a wealth of mitochondrial genome (mitogenome) sequences that could benefit several fields, including population studies, disease association studies and forensic genetics. In addition, donor metadata is often available since WGS researchers are interested in associations between genetic information and individual characteristics. These metadata, such as geographic origin, ancestry, metapopulation, phenotype, age and sex, may also be useful in mitochondrial DNA (mtDNA) research. WGS studies typically involve a large number of samples that are sequenced using next-generation sequencing (NGS) technologies and high-throughput instruments to make the study more cost-effective. By using already produced and publicly available WGS data, the initially ignored mitogenome analysis can be performed with no processing costs.

However, nuclear mtDNA segments (NUMTs) from chromosomal DNA may prevent the ability to produce reliable mitogenome haplotypes from untargeted (or shotgun) WGS reads. NUMTs are portions of mtDNA that have been inserted into the nuclear genome, most of them within the last 100,000 years [2]. There are more than 1090 documented NUMTs to date, ranging in size from 39 to 18,649 base pairs (bp), including three that are larger than the entire mitogenome (16,569 bp) [3,4]. In addition, the existence of entire copies of the mitogenome that were tandemly inserted as many as 50 times into a single location in the nuclear genome (referred to as a “mega-NUMT” by [5]) has been recently reported [6]. Though mtDNA is generally more abundant than nuclear DNA in human cells, the relative proportions of the two genomes (i.e., mitochondrial and nuclear) vary across sample types [7], and will therefore impact the prevalence of NUMTs in mtDNA alignments. NUMT reads that map to the mitochondrial genome will be observed as mixed at positions where the NUMT and authentic mtDNA haplotypes differ, but mixed positions also occur when the mtDNA molecules within a sample vary due to heteroplasmy.

NUMT variants are often difficult to distinguish from authentic heteroplasmy and other sources of mixed data such as contamination from exogenous mtDNA, PCR or sequencing errors, and DNA damage (e.g., cytosine deamination) [4]. This is especially true when applying low variant detection thresholds. Based on previous high-quality mitogenome studies [8,9], no more than three point heteroplasmies (PHPs) are expected in a human mitogenome per individual when employing a minimum 5–10% variant detection threshold. Therefore, haplotypes that contain numerous and/or closely located PHPs are unexpected and require critical review. The ability to correctly differentiate between authentic heteroplasmy and NUMTs, or other sources of mixed data [4], is essential. Many studies rely on low-level variants (heteroplasmy) for disease association and population studies (e.g., [10,11,12,13]). If NUMTs or other artifacts are erroneously included in the mtDNA haplotypes, the results may lead to unsupported conclusions [14,15,16]. NUMTs were misinterpreted as paternal mtDNA in a study published in 2018 [17]. The authors questioned the long-standing inheritance pattern of mtDNA, but received critical comments [18,19]. The problem with NUMTs in mtDNA data is not restricted to human studies, as concerns about NUMT contamination have also been documented in mtDNA research involving other species (e.g., [20,21,22]).

To prevent NUMTs from interfering with mtDNA alignments, targeted mtDNA enrichment can be performed before sequencing using long-range amplification [23] and other approaches [24]. However, large NUMTs and mega-NUMTs may still be co-amplified, but they are rarely observed in the general population. Other enrichment approaches used for degraded DNA samples, such as small-amplicon and hybridization capture methods, are also known to produce NUMT reads in the NGS data [23,25,26]. Thus, NUMTs cannot be entirely avoided in mtDNA analyses. For this reason, specialized analysis tools have been developed to prune variants associated with NUMTs from the haplotype. Ring et al. [23] utilized alternative mapping strategies, including increased stringency, competitive mapping to the human reference genome (i.e., nuclear and mitochondrial genomes), and stringent alignment to the sample consensus sequence to prevent the mapping of NUMT reads based on the dissimilarity between the NUMT and mtDNA sequences. Other approaches to minimize NUMT reads in mtDNA mappings include filtering the sequences found in a catalog of known NUMTs, which is applied by the Verogen Universal Analysis Software [27] and the Remove the NUMTs! (RtN!) open-source application [3]. Another method for minimizing NUMT interference is to perform a manual pruning of variants that are known to be associated with NUMTs [26,28]. This can also be done automatically, but may also result in the removal of authentic PHPs [23]. Furthermore, the ability to distinguish between NUMT and mtDNA reads often relies on the phasing of clustered variants [4]. Although detectable in NGS data, unlike Sanger sequencing data that would require cloning to parse out individual DNA sequences, variant phasing may be invisible in short-read data (e.g., 35–75 bp reads) depending on the distance between the variants associated with NUMT reads [4,29]. To ensure that NUMTs are not reported in the mtDNA haplotype, a combination of approaches (e.g., enrichment, mapping, phasing assessment, and variant pruning) may be required.

The present study evaluated the ability to produce authentic mtDNA haplotypes from WGS using the pre-existing SweGen dataset [30]. This WGS dataset is comprised of 942 genomes from unrelated Swedish individuals, which were selected based on microarray data from the TwinGene project [31] to represent a cross-section of the Swedish population. Extensive genetic variation analyses were performed based on the nuclear data [30], but, like many WGS studies, the mitogenomes were not previously examined. The study will identify analysis parameters necessary to overcome NUMT interference in WGS data to generate high-quality mitogenome haplotypes from existing data and thus at a low cost.

## 2. Results

### 2.1. Overall Performance

#### 2.1.1. Passing Samples

The mitogenome data from the WGS of 942 Swedish individuals were analyzed in this study. In total, 917 samples generated mitogenome haplotypes that were determined to be of high quality based on two independent reviews and a rigorous quality control (QC) process (Table 1). These SweGen haplotypes were categorized as “passing” all QC criteria and thus included in the final dataset (Table S1). There were 858 samples that produced complete 100X read depth across the entire mitogenome, averaging 2365X and ranging from 502X to 1050X (Table 1). The minimum read depth of 100X, which is relatively high for WGS data, was utilized in this study to ensure that low mtDNA coverage would not complicate the assessment of NUMTs. There were 59 additional haplotypes that were considered passing despite having up to four positions below the 100X threshold. Read depths below this threshold were observed at eight positions in or surrounding polycytosine (C-) stretches, particularly the C-stretch in the hypervariable segment 2 (HVS2) region: nps 302 (50), 301 (12), 310 (11), 567 (8), 10946 (7), 297 (1), 455 (1), and 16182 (1). The reduced read depths were due to indel misalignment in these homopolymeric regions, which were worsened by overall reduced read depths (averaging 653X) in these haplotypes (Table 1). Across these 59 nearly complete mitogenomes, 91 positions below the threshold were included in the mtDNA haplotypes, with minimum read depths for each sample ranging from 47X to 97X (Table S1). In addition, 25 samples were excluded based on the specific QC criteria (Table 1), and these data are discussed in detail below (see Section 2.1.2).

**Figure 1:**
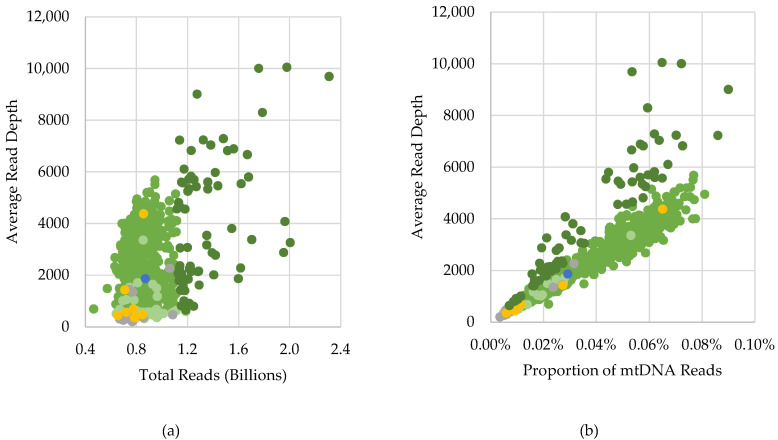
Scatterplots of the correlation between (**a**) total reads (billions) and (**b**) the proportion of mapped reads that mapped to the mtDNA reference genome and the average mtDNA read depth. Samples are plotted based on the category and subclassification: green = complete with less than 1.15 billion total reads (*n* = 785), dark green = complete with more than 1.15 billion total reads (*n* = 73), light green = nearly complete (*n* = 59), gray = incomplete (*n* = 17), yellow = mixed (*n* = 7), and blue = related (*n* = 1).

Overall, passing mtDNA data were generated from samples with as few as 464 million total WGS reads. Most passing haplotypes (> 93%) were produced from WGS data with 518 million to 1.15 billion reads, averaging 864 million total WGS reads per sample (Figure S1). Though the percentage of total reads initially mapped to GRCh37 averaged 99.2% for the passing samples (Table 1), only a small proportion of the mapped reads aligned to the revised Cambridge Reference Sequence (rCRS) [32,33] in the mapping used for mtDNA analysis (0.005–0.090%, average of 0.034%). The proportion of mtDNA reads, rather than the total reads (Figure 1a), correlated with the average read depth observed (R^2^ = 0.9370 excluding samples with more than 1.15 billion reads; Figure 1b) and ultimately impacted the acceptability of the mtDNA data. The proportion of mtDNA reads is representative of the relative mtDNA copy number within a sample or the ratio of mtDNA copies to nDNA copies. It is therefore not unexpected that fewer mtDNA copies present in a sample would result in reduced coverage of the mitogenome from untargeted sequence data. However, as a result of the large number of WGS reads, passing mitogenome haplotypes were obtained, with sufficient depth and breadth of coverage for nearly all (917; 96.7%) SweGen samples.

#### 2.1.2. Excluded Samples

There were 25 of the 942 SweGen samples that did not meet the QC criteria set forth for the dataset and therefore were excluded from analysis in this study (Table 1). These excluded samples were incomplete haplotypes (>four positions below the 100X threshold), possible mixtures, or maternal relatives of another SweGen sample (Table S1). A majority (68.0%) of the excluded haplotypes were incomplete. As noted above, the 100X minimum read depth threshold was utilized to eliminate low coverage data from impacting the NUMT assessment. The minimum read depths for these samples ranged from 6X to 79X, with all except one with 20X or greater minimum coverage. Although WGS generated an average of 784 million reads for the 17 incomplete samples, most (82.4%) had average read depths below 500X, corresponding to low proportions of mtDNA reads (<0.008%; Figure 1b). Fourteen of the incomplete haplotypes had an average of 12 positions (ranging from five to 25 positions) that did not have sufficient coverage depth. The three other incomplete samples had 70 or more positions below the 100X threshold. The sample (SWE918) with the most (114) positions below the threshold also had the lowest average read depth (201X) and mtDNA proportion (0.003%) (Table S1). Many of the incomplete haplotypes had numerous (as many as 13) mixed positions detected above the 5% frequency threshold, which may be the result of NUMTs, and, therefore, further complicated the analyses of these low coverage data. However, a majority (71; 77.2%) of the 92 mixed positions observed in the incomplete samples had a minor nucleotide represented by less than 10% of the reads. Moreover, most (17; 80.9%) of the 21 mixed positions observed at a 10% frequency or higher were clearly identifiable NUMT variants (Table S1). Therefore, it may be possible to utilize a lower read depth threshold (e.g., 20X) if the variant detection threshold was increased, which would allow the inclusion of the full mitogenomes of these “incomplete” samples in other studies. There were three incomplete mitogenome sequences with relatively high average read depths and mtDNA proportions (>1300X and >0.022%, respectively) that had no indication of NUMT interference. These three incomplete profiles were missing 11–12 positions in the HVS2 C-stretch. Interestingly, all three haplotypes belong to mtDNA haplogroup J1c2e2 (Table S1), which has five diagnostic variants between np 185 and np 295 (G185A, A188G, G228A, A263G, C295T based on the rCRS-oriented version of Phylotree Build 17 [34]). Inspection of the GRCh37 BAMs for these samples identified a dramatic loss in reads that extended across the region preceding the HVS C-stretch compared to other samples. Though 18 passing haplotypes were assigned to J1c2 haplogroups, which also had these five variants, mapping stringency combined with poor sequencing through the HV2 C-stretch may explain the regional loss in coverage for these three samples.

Seven complete or nearly complete mitogenome haplotypes were flagged as possible mixtures, with as many as 35 mixed positions detected with the 5% frequency threshold. Of the 146 mixed positions observed in these samples, less than one-third (41; 28.0%) would be observed with the use of a 10% frequency threshold (Table S1). Thus, with the use of the higher frequency threshold, it may be difficult to detect these mixtures in the mtDNA, with the exception of one sample (SWE939) that had 34 mixed positions above 10% (35 were detected with the 5% threshold). Low-level (<10–20%) mixtures are not easily detectable in nuclear SNP data, especially when the samples are expected to originate from a single source. As a result, these seven samples were included in the nuclear analyses described in Ameur et al. [30]. Five of the seven possible mixtures had relatively low proportions of mtDNA (<0.012%) and correspondingly low average read depths (<700X).

In these particular samples, it was difficult to confidently determine the source of the mixed positions as contamination from an exogenous DNA source (i.e., another sample) or from NUMT reads because the mixed positions were found throughout the mitogenome, including haplogroup diagnostic sites. Unfortunately, confirmation testing using an enrichment method such as long-range amplification to minimize NUMT interference was not possible for the SweGen dataset. In these cases, the mtDNA haplogroup of the major and minor haplotypes may provide some guidance to the source of the mixture (Table S1) [35,36], and potentially assist in the identification of NUMTs [37]. However, phylogenetic analysis does not eliminate the possibility that the mixture results from a mega-NUMT from a “modern” mtDNA haplogroup, as was detected in Lutz-Bonengel et al. [6].

The last sample excluded from analysis shared a complete mitogenome haplotype with another SweGen sample that was identified as a first-degree maternal relative (kinship coefficient of 0.2898 based on nuclear data [30]). Often, supplemental nuclear DNA typing must be performed to assess relatedness for samples with shared mtDNA haplotypes within a population dataset. The existing WGS data benefitted the SweGen mitogenome analysis since nuclear genetic analyses had already been performed. The ability to use the WGS results for kinship analysis saved time and resources that are typically required to complete this important QC measure for mtDNA population studies [38].

### 2.2. Classification of Mixed Positions

**Figure 2:**
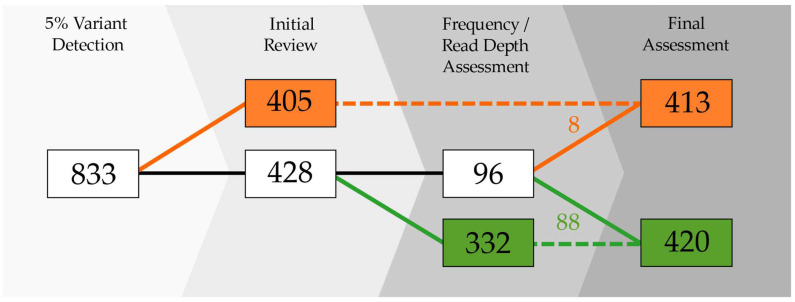
Classification of mixed positions that were observed in the mitochondrial genome (mitogenome) haplotypes from the SweGen whole-genome sequencing data. Mixed positions (white) were identified during initial variant detection using a 5% minimum minor nucleotide (light gray). These 833 mixed positions were detected with a 5% minor nucleotide frequency threshold and classified as either nuclear mtDNA segment (NUMT) variants or point heteroplasmies (PHPs) during multiple assessments (gray gradient). The 413 NUMT variants are shown in the inner plot (orange; scale 0–60 observations) and the 420 PHPs are displayed in the outer plot (green; scale 0–35 observations).

**Figure 3:**
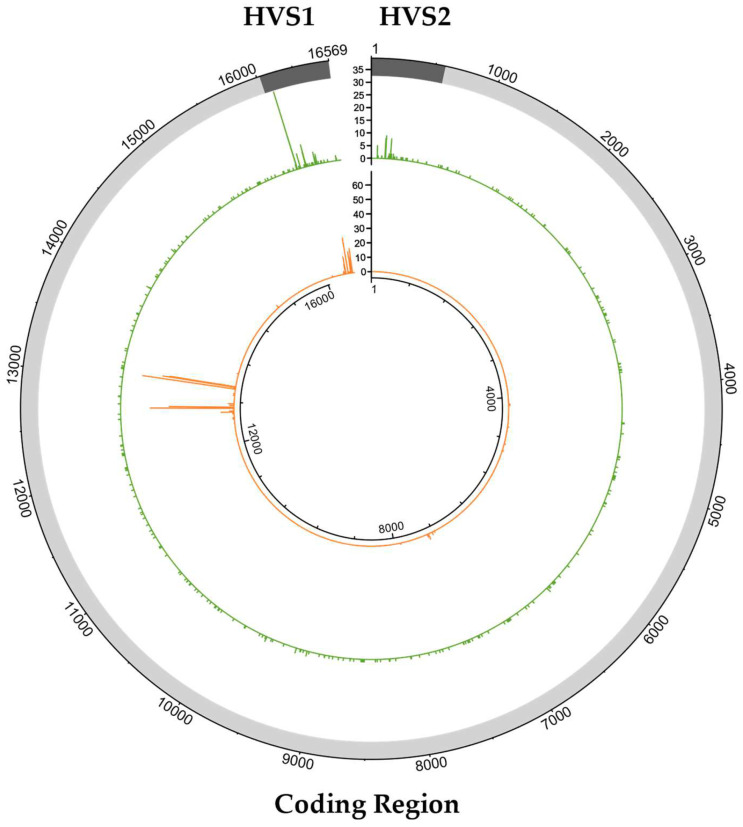
Distribution of mixed positions across the circular mitogenome, including the hypervariable segments 1 and 2 (HVS1 and HVS2, respectively; dark gray) of the mtDNA control region and the entire mtDNA coding region (light gray). These 833 mixed positions were detected with a 5% minor nucleotide frequency threshold and classified as either a NUMT variant or PHP. The 413 NUMT variants are shown in the inner plot (orange; scale 0–60 observations) and the 420 PHPs are displayed in the outer plot (green; scale 0–35 observations).

**Figure 4:**
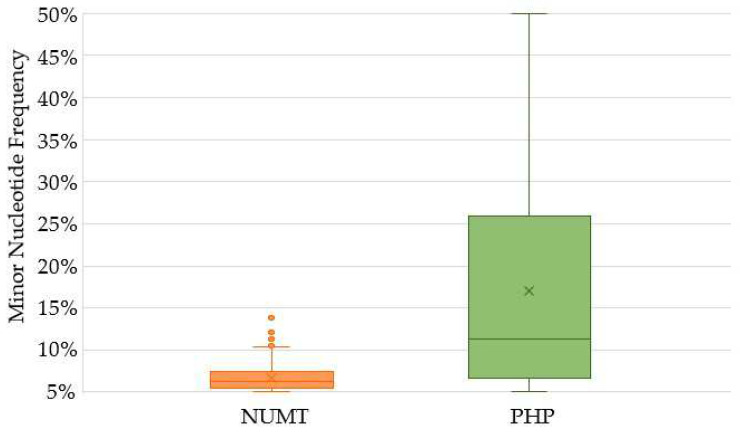
Distribution of the frequency (>5%) of the minor nucleotide for the 420 PHPs (green) included in the SweGen haplotypes and the 413 NUMT variants (orange) identified in the mitogenomes. The av-erage for each classification is shown as an “×” and outliers are shown as single data points.

The data were first analyzed at the 5% variant detection threshold that has been previously shown to be appropriate for high-quality mtDNA haplotype generation [8]. When the 5% frequency threshold was applied, there were 833 mixed positions detected in the 917 passing mitogenome haplotypes (Figure 2). During the initial review, nearly half (405; 48.6%) of the variants were attributed to NUMT reads by the two reviewing analysts. The identification of NUMT variants during this initial review was based on mixed positions observed in clusters, coding region (codR) hotspots (i.e., mixed positions observed in multiple samples), association with other heteroplasmic variants detected at the 2% frequency threshold, and phasing in the read mappings. Nearly 95% (383) of the 405 NUMT variants were isolated to two mtDNA regions: nps 12612–13105 and 16390–16527 (Figure 3). The most common NUMT variants were A13062G (65), G12684A (58), T13095C (51), C12705T (45), and A13105G (46). In the hypervariable segment 1 (HVS1) region, C16444T (25) was frequently identified as a NUMT variant during the initial review, followed by C16527T (17) and G16496A (15). More than 95% of the 405 NUMT variants initially identified were observed at frequencies less than 10%, and the maximum frequency was 14.3% (Figure 4).

**Figure 5:**
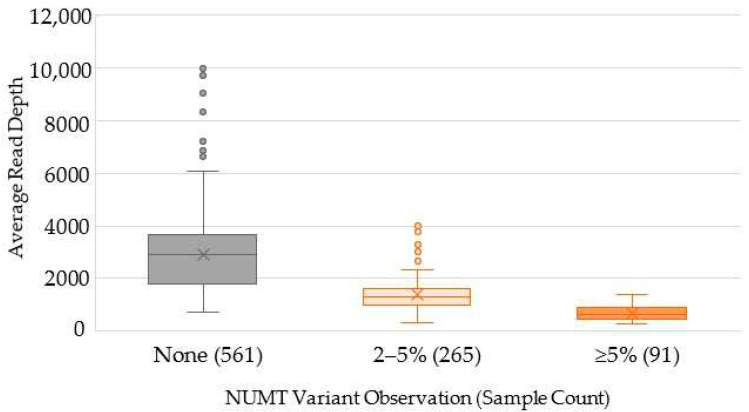
Distribution of average read depth based on the detection of NUMT interference at either a 2% frequency threshold or 5% frequency threshold. The sample count per NUMT variant observation category is noted in parentheses for each category. The average for each classification is shown as an “×” and outliers are shown as single data points.

The 428 mixed positions remaining in the haplotypes after the initial NUMT review were further assessed (Figure 2). Since NUMT variants were found to be present in samples with average read depths below 1500X (Figure 5) and primarily at frequencies <10% (Figure 4), these two criteria were utilized in the secondary review of the mixed positions to further classify additional NUMTs. The secondary assessment identified 96 mixed positions that met these criteria (<10% frequency of the minor nucleotide and average read depth ≤1500X). These 96 mixed positions were assessed by performing a review of other mixed positions in the sample, comparison with known PHP hotspots [8,39], comparison with known NUMT variants (e.g., documented in [25], identified as NUMTs during the initial NUMT review), and inspections of low-frequency variants (2–5% minor nucleotide frequency) and the read mapping. For a majority (91.6%) of the 96 evaluated heteroplasmic variants in this assessment, NUMT interference was not suspected; though, the short reads often prevented a proper determination of variant phasing. In the end, eight additional NUMT variants were identified in the haplotypes from the 96 mixed positions, three of which were detected in one sample (C4155, A4917, and G5054) and two in another sample (G5147 and G14905).

After multiple NUMT assessments of the mitogenome data using the 5% frequency threshold, 413 mixed positions were associated with NUMTs in 91 of the SweGen samples (Table 2). The remaining 420 mixed positions could not be associated with a NUMT and were therefore classified as probable PHPs (Table S1).

### 2.3. Characterization of Variants Associated with NUMTs

Of the 917 SweGen samples with passing mitogenome haplotypes, 356 (38.8%) contained detectable NUMTs in the SweGen WGS data when the 2% frequency threshold was applied (Table 2). As discussed previously, NUMT interference was more frequent in samples with low average read depths. In fact, only 74 (13.2%) of the 561 samples in which no NUMT interference was observed had average read depths less than 1407X, which was the maximum read depth for samples with NUMT variants that were detected above the 5% frequency threshold (Figure 5). Consequently, due to the strong correlation between the average read depth and mtDNA proportion (Figure 1b), fewer mtDNA reads relative to nuclear reads increased the observation of NUMT variants, which were derived from the latter (i.e., nuclear reads).

The 413 NUMT variants detected with the 5% frequency threshold were identified at 47 positions across the mitogenome (Figure 3, Table S2). The majority (33; 70.2%) were located in the codR (nps 577–16,023), and the remainder was detected in the control region (CR; nps 16,024–576). There were 383 (92.7%) NUMT variants in the two previously noted NUMT hotspot regions (nps 12,612–13,105 and 16,390–16,527). Additional NUMT hotspots were observed at nps 7220, 7229, and 7325. At each of the 47 positions that were impacted by NUMT interference, one variant was observed as a result of NUMT reads (i.e., 47 different NUMT variants). Most (28) of the 47 observed NUMT variants were consistent with the rCRS nucleotide (i.e., here, the sample mtDNA differed from the rCRS), but these rCRS “variants” only represented 48 (11.6%) of the 413 NUMT variants. The remaining NUMT variants (19) were transitions relative to the rCRS, and these variants were observed more frequently (365 of 413 total NUMT variant observations). However, only 13 of the 47 NUMT variants identified in this study were documented since NUMT variants consistent with the rCRS were not reported by Li et al. [25]. Li et al. [25] presented a list of more than 10,000 variants associated with NUMTs in contrast to the NUMT sequences described in other studies (e.g., [2,3]), making this NUMT variant catalog easy to use for the characterization of mixed positions. However, the list is unlikely to be comprehensive and thus may not include all possible variants.

The six non-rCRS NUMT variants observed in the 5% frequency threshold data that were not documented in the study by Li et al. [25] include the following: A13062G, G16129A, G16390A, A16399G, C16444T, and G16496A. These six variants accounted for nearly one third (125/413) of the observed NUMT interference. Interestingly, A13062G was not documented in Li et al. [25] but was always observed in-phase with Li et al. NUMT variants located nearby (i.e., T13095C and A13105G) (Figure S2a). When a 51-bp sequence (nps 13,060–13,110) with these three presumed NUMT variants (i.e., A13062G, T13095C and A13105G) was searched in BLAST [40], seven identical sequences (100% query coverage) were identified (Table S3). This presumed NUMT sequence observed in the SweGen dataset was consistent with polymorphic NUMT sequence HGDP01029 [2] as well as one Neanderthal and five Denisovan mitogenomes, confirming the source of these variants as NUMTs. Four of the undocumented Li et al. NUMT variants were observed in reads that included six in-phase variants: G16390A, A16399G, C16444T, G16496A, T16519C and C16527T (Figure S2b). This 156-bp sequence (nps 16,380–16,535) was searched in BLAST [40], and five matching sequences were identified (Table S4). Four of the matches were NUMTs, two of which were previously published (HGDP00856 [2] and HSA_NumtS_587 [41]) and two others that were submitted to GenBank without an associated publication. The fifth matching sequence was a Filipino complete mitogenome (AgI4055) produced with hybridization capture enrichment and Illumina sequencing [42]. This haplotype was generated from relatively low coverage data (average 85X) compared to other samples in the study and included positions covered by a single read [42], which may have resulted in the inclusion of a NUMT sequence in this mtDNA haplotype (nps 16,054–16,569, 1–73). Again, especially for low coverage WGS or capture data, a phylogenetic review (i.e., mtDNA haplogroup prediction) would likely provide a valuable QC check of the mitogenome haplotype [35,36,43].

With the exception of four outliers, the frequency of NUMT variants did not exceed 11.5% (Figure 4). In fact, only 13 (3.1%) of the 413 NUMT variants would be detectable with a 10% frequency threshold. These were observed exclusively in the NUMT hotspot region located at nps 12612–13105 (Table S2). Although reduced average read depths showed an increase in NUMT interference (Figure 5), the average read depth showed no correlation (R^2^ = 0.0979) with the minor nucleotide frequency of NUMT variants (Figure S3a). The two NUMT variants with the highest minor nucleotide frequencies (~14%) were detected in a sample (SWE859) with the lowest average read depth (318X) of the passing haplotypes. This sample also had the highest number of NUMT variants (*n* = 14). Generally, the number of NUMT variants detected above the 5% frequency threshold in a sample tended to increase, as the average read depth decreased (Figure S3b). Since there was a correlation between average read depth and the mtDNA proportion, the same trends were also observed between the proportion of mtDNA reads and the NUMT nucleotide frequency (Figure S3c) and a number of NUMT variants observed (Figure S3d). Furthermore, the eight samples with NUMT variants observed at a 10% frequency or higher had average read depths less than 550X and very low mtDNA proportions (<0.01%).

### 2.4. Heteroplasmy

In contrast to the average minor nucleotide frequency of 6.7% for the variants detected at a 5% threshold that was associated with NUMTs, the average frequency of the minor nucleotide of PHPs was much higher at 17.0% (Figure 4). Furthermore, the minor nucleotide frequency for half of the PHPs was more than 11.2%, whereas all except four NUMT variants had minor nucleotide frequencies less than 11.5% (Figure 4). There were 185 PHPs with minor nucleotide frequencies less than 10% and an additional 68 PHPs with minor nucleotide frequencies less than 15%. Above the 15% minor nucleotide frequency, the number of PHPs that were observed ranged from 15 to 32 for each 5% frequency window (i.e., 15–20%, 20–25%, etc.) and totaled 167 PHPs altogether (Figure S4).

The 420 PHPs detected at the 5% frequency threshold were observed at 322 positions (Figure 3), with 64 positions in the mtDNA CR and 258 positions in the mtDNA codR. Although there were four-fold more positions in the codR with PHPs, a larger proportion of the CR positions were heteroplasmic compared with the codR (5.7% vs. 1.7%, respectively). There were 156 PHPs observed in the CR, with a maximum of 32 observations at one position (np 16,093). Other heteroplasmic hotspots were observed at nps 152 (9), 16192 (9), 146 (8), and 204 (8).

Conversely, more PHPs were detected in the codR (264), but with only one or two observations per codR position (Table S5). There were 22 PHPs in homopolymer regions across the mitogenome including 20 in the HVS1 C-stretch (at nps 16,188, 16,189, 16,192, 16,193 and 16,195), one at np 316 at the end of the HVS2 C-stretch, and one at np 955 at the beginning of another C-stretch at nps 956–960. Most (406; 96.7%) of the PHPs were transitions (241 C/T and 165 A/G) and only 14 heteroplasmies were transversions (five A/C, four A/T, three G/C, and two G/T). Overall, the minor nucleotide at heteroplasmic positions was most often a variant (317; 75.5%) from the rCRS nucleotide, although one-fourth of the PHPs were observed with the rCRS base as the minor nucleotide (Figure S4).

In the SweGen mitogenome dataset, 334 of the 917 passing haplotypes (36.4%) had at least one PHP when the 5% frequency threshold was applied (Table S1). Most (263; 78.7%) of these only had one PHP, while 57 haplotypes had two PHPs and 13 haplotypes had three PHPs. A single haplotype had four PHPs, the maximum number of heteroplasmies that were observed among the SweGen mitogenomes. This sample (SWE777) had an average read depth exceeding 4000X with a relatively high proportion of mtDNA (0.056%). Thus, the haplotype was not suspected to be impacted by NUMT interference (Figure 5), yet two of the variants (T11386C and T12285C) were associated with NUMTs by Li et al. [25] (Table S2). There were over 100 PHPs (≥5%) that were Li et al. NUMT variants, but most were observed once or at known heteroplasmic hotspots [39]. Furthermore, the four PHPs were observed at varying minor nucleotide frequencies (C2887Y 31.2%, T11386Y 9.5%, T12285Y 33.7%, and T16192Y 11.4%). With the application of a 10% frequency threshold, one of the two variants associated with NUMTs by Li et al. would be removed. Overall, when a 10% threshold is applied to the SweGen PHPs, the proportion of haplotypes with PHPs was reduced to 22.3%, with a maximum of three PHPs observed in a single haplotype (Table S5). However, there were still 69 PHPs detected with the 10% frequency threshold that were associated with NUMTs by Li et al. [25], including common PHPs T146Y, T152Y and T16311Y (Table S2).

There were five PHPs that were also classified as NUMT variants in other samples. During the multiple assessments, these PHPs were scrutinized for variants caused by NUMTs. Phasing, NUMT variant observations in the sample, average read depth, and mtDNA proportion were all considered in the assessment. After this thorough review, G16129A (at 5.4%) was classified in one sample as a NUMT variant (SWE866), but G16129R was included in five haplotypes as a PHP with A as the minor nucleotide across difference haplogroups (Table S1). Classification at this position is complicated since G16129A is a diagnostic site for many haplogroups [34] and is also a known heteroplasmic hotspot [39]. The PHP A13105R (G at 15.3%) was maintained in one sample (SWE658) due to its high average read depth and mtDNA proportion (3287X and 0.053%, respectively), despite the fact that A13105G was classified as a NUMT variant in 46 other samples (Table S2). The remaining three variants classified as both a PHP and NUMT variant in the 5% frequency data were C16354, G16390A and T16519C (Table S2). In contrast, no PHP was also classified as a NUMT variant when the 10% frequency threshold is applied, substantially simplifying the classification of mixed positions observed in the WGS mtDNA data.

### 2.5. Comparison with High-Quality Mitogenome Datasets

Considering the 5% frequency threshold data, there was a notable difference between the proportion of samples with PHPs in SweGen dataset (36.4%) compared to the platinum-quality NGS mitogenome dataset of Taylor et al. (27.8%) [8]. The increased PHP frequency in the SweGen data was primarily due to a higher proportion of samples with one PHP than the Taylor et al. dataset. Additionally, the maximum number of PHPs per individual in the SweGen data was four (SWE015) compared to only three in the Taylor et al. mitogenomes. Though the overall PHP distributions across the mitogenome were similar between the two 5% threshold datasets, there were more CR PHPs in the SweGen mitogenomes than in platinum-quality data [8] (Table S5). In particular, there was a considerable increase of T16093Y occurrences in the SweGen data (*n* = 32) compared to ten T16093Y PHPs among the 1327 Taylor et al. mitogenomes. To eliminate dissimilarities in haplogroup distribution as a possible reason for this difference, variation at np 16093 in the two U.S. Caucasian populations of Taylor et al. were further investigated. Both the SweGen and Taylor et al. U.S. Caucasian datasets had approximately 7% of haplotypes with either a T16093C or T16093Y (Figure S5), indicating similar frequency in the two datasets. However, the proportion of haplotypes with heteroplasmy (Y) compared to the homoplasmic variant (C) at np 16093 was increased in the SweGen datasets (49.2% versus 23.1% in the U.S. Caucasians). Furthermore, most (81.3%) of the SweGen T16093Y PHPs had the rCRS thymidine (T) as the minor nucleotide, whereas the C and T were represented in equal proportions as the minor nucleotide in the U.S. Caucasian data (Figure S5).

The U.S. Caucasian data of Taylor et al. [8] were generated with long-range amplification, a method considered to effectively eliminate NUMT interference. In contrast, the overrepresentation of T16093Y in the SweGen mitogenomes, specifically those haplotypes with the rCRS T as the minor nucleotide, were produced from short-read WGS data and may be the result of NUMTs. For example, the NUMTs with significant homology to the mtDNA HVS1 that were identified through a BLAST search (Table S4) were consistent with the rCRS at np 16093. It is, therefore, possible that a portion of the T16093Y PHPs with the rCRS T as the minor nucleotide is in fact, NUMT variants and should not be included in the mtDNA haplotype. Of the 26 SweGen haplotypes with the rCRS T as the minor nucleotide in the T16093Y, 12 samples had lower average read depths (<1500X) combined with low minor nucleotide frequencies (<10%) (Table S1). These 12 samples were therefore included in the further in-depth NUMT assessment (Figure 2). However, only two of the 12 samples had NUMT variants in HVS1 (e.g., G16390A, A16399G, etc.) detected with the 5% frequency threshold.

Additionally, the short read lengths of the SweGen WGS data made it impossible to assess phasing in the read mapping between the T16093 and NUMT variants observed in HVS1. Classification of PHPs or NUMTs is further complicated, as np 16093 is a known heteroplasmic hotspot [39] with variations observed across sample types [44]. Cihlar et al. [26] have previously discussed the difficulty of distinguishing authentic heteroplasmy at np 16093 from NUMT variants in small amplicon NGS data, and suggested the use of phylogenetic information as a possible solution [37]. Another, more conservative option for a hotspot like np 16093 could be to ignore the position in all data interpretations due to its variability and susceptibility to NUMT interference. However, even when T16093Y is ignored in the 5% threshold datasets, the proportion of SweGen individuals with PHPs was still elevated (34.1%) compared to the Taylor et al. data (27.1%) (Table S5).

To assess the utility of a higher frequency threshold for mitogenome analysis of WGS data, 10% threshold data were compared across the SweGen, Taylor et al. [8], and Just et al. [9] datasets (Table S5). Of particular note, only six (18.8%) of the 32 T16093Y in the 5% threshold SweGen data would be detectable with the 10% frequency threshold. As a result of the 10% frequency threshold, the proportion of individuals with PHPs was comparable between the SweGen and Taylor et al., though still slightly (3.9%) higher in the SweGen samples (Table S5). All PHP results were strikingly similar between the SweGen 10% threshold data and the Just et al. Sanger dataset. NUMT interference in the Just et al. mitogenome data was possible due to the size and design of the amplicons (~2500 bps) [9,45]. However, the Just et al. dataset was produced from serum, which contains primarily circulating cell-free DNA since nucleated cells are removed. The cell-free nuclear DNA in serum has been shown to be less intact than the cell-free mtDNA [46], and thus NUMT co-amplification would be minimized with this sample type and amplification approach.

As an additional evaluation, the Li et al. blood and skin specimen data [11] at both 5% and 10% thresholds were also compared to the SweGen data (Table S5). The PHP results in the Li et al. datasets were similar to the SweGen data when using both frequency thresholds, and the 10% data were also consistent with the Just et al. Sanger dataset. However, Li et al. employed a capture enrichment method and it may, therefore, be prone to NUMT interference like the SweGen WGS data [4]. Ultimately, enrichment with long-range amplification is likely to be necessary to classify mixed positions as either authentic PHP or an artifact of NUMTs with high certainty, as demonstrated in other studies [23,47,48].

## 3. Discussion

A valuable QC metric for the SweGen WGS data was the average read depth, as it provided an indication that NUMTs may interfere in the mtDNA analysis. Though samples with greater than 1500X average read depth had no observed NUMT variants (Figure 5), this value may not be applicable for other datasets because many factors can impact the coverage. Different WGS approaches, and specifically, the choice of sequencing platform and the multiplexing level, can significantly affect the number of total reads per sample and consequently the read depth. In this study, both the average read depth *and* the proportion of mtDNA reads were shown to have inverse relationships with the observation of NUMT interference (Figure S1d). The proportion of mtDNA reads to nuclear DNA reads (i.e., relative mtDNA copy number) is likely to offer a more consistent QC metric applicable across WGS datasets, if available, rather than coverage depth. However, a study by Singh et al. [49] did not detect a correlation between NUMT interference and mtDNA proportion of WGS reads, but the authors acknowledge the small sample size (*n* = 9) and minimal variation of mtDNA copy number as a possible explanation for their findings. The Singh et al. study did show an overall increase in heteroplasmy error with reduced read depths when analyzing mtDNA reads from WGS data [49], which indicates that read depth is still a useful QC metric and the importance of a sufficient minimum coverage threshold. Nevertheless, the mtDNA proportion may be more suitable to utilize as a means to identify samples prone to NUMT interference since the proportion of WGS reads aligned to the mtDNA reference provides an assessment of the mtDNA copy number relative to the nuclear genome in an individual [50,51,52].

In more than half (56) of the 91 samples with NUMT interference observed above the 5% frequency threshold, numerous (≥4) low-level variants were clustered in known NUMT hotspot regions (e.g., nps 12612–13105). This allowed the NUMT variants to be easily identified during the initial review. However, several haplotypes (19) had only one NUMT variant, often along with other mixed positions that were maintained as probable PHPs. Generally, these single NUMT variants were reoccurring at certain positions within this dataset (e.g., np 13062), which made the identification of NUMTs easier. The NUMT variant classification was also facilitated by the assessment of phasing in the read mapping and further review of variants detectable with the 2% frequency threshold. The ability to evaluate variants above the background noise, but below the frequency threshold used for analysis (e.g., 5% or 10%), can provide valuable information about the quality of the mtDNA profile. This may be especially important for the detection of low-level mixtures, which may not be easily identified with a higher frequency threshold. Though some mixed positions were readily classified as NUMTs or PHPs, T16093Y posed the greatest challenge. Due to its unstable nature [39,44], heteroplasmy at np 16093 is expected and neither T16093 nor T16093C was associated with NUMTs by Li et al. [25]. And though the rCRS nucleotide (T) is consistent with NUMTs commonly observed in the HVS1 region of the SweGen mtDNA data (e.g., [2,41]), the short read lengths prevented the assessment of phasing. A potential option for heteroplasmic hotspots and positions highly susceptible to NUMT interference (e.g., nps 16093 and 13062) could be to ignore these positions in all data interpretations, similar to the treatment of length heteroplasmy (LHP) indels [53].

The ability to distinguish between authentic point heteroplasmy and NUMT variants can be complicated at certain positions, especially in positions where PHPs are known to occur. It may, however, be possible to implement bioinformatic steps and/or modify analysis parameters to minimize NUMT interference, thus reducing the need for the detailed reviews implemented in the present study. For example, mtDNA analysis pipelines for WGS data may incorporate NUMT read filtering such as the RtN! tool [3]. However, RtN! and other software that implement similar approaches (e.g., the Converge Forensic Analysis Software from Thermo Fisher Scientific [28] and Verogen’s Universal Analysis Software [27]) were developed for small amplicon enrichment data rather than WGS data. The assessment of phasing is more straightforward for enrichment methods that utilize small amplicons since the whole amplicon is sequenced in each read. Moreover, the expected NUMTs that may be co-enriched by known amplicons can be more easily characterized and flagged than those that may be observed in unenriched WGS data. Promising mtDNA results from WGS data have been produced with the recently-introduced MitoScape software, which applies machine learning to model NUMTs [49]. Development of such software designed to analyze mtDNA analysis of WGS data, and specifically addressing NUMT interference, can minimize the need for exhaustive data review in order to produce reliable mitogenome haplotypes.

As an alternative to the bioinformatic tools that eliminate the NUMT reads from the mtDNA alignment, adjustments to the variant detection process can also reduce the impact of NUMT interference. Pruning that is based on a catalog of known NUMT variants can provide automated removal [23], but this strategy requires a comprehensive and carefully curated catalog of known variants for complete removal of NUMT interference from the mtDNA haplotypes. For example, approximately 40% of the mixed positions classified as NUMT variants in this study would remain in the sample haplotypes if utilizing the more than 10,000 variants associated with NUMTs by Li et al. [25]. This approach may not be preferred, as novel NUMT variants would not be pruned and authentic PHPs could be removed [23]. Another option may be to increase the minimum variant detection threshold. For example, the use of an elevated 10% frequency threshold eliminated nearly all NUMT interference (400/413; 96.9%); however, almost half (44.5%) of the presumably authentic heteroplasmic variation would also be eliminated. The 10% frequency threshold for the SweGen WGS data would substantially reduce the review burden, requiring edits to only eight samples due to NUMT interference instead of 91 samples with the 5% frequency threshold.

Additionally, the proportion of SweGen samples with PHPs using the 10% frequency threshold was more similar to other high-quality mitogenome datasets [8,9]. Depending on the application of the mtDNA data, researchers need to evaluate the implication of including artifacts (i.e., variants associated with a NUMT) versus missing heteroplasmic variation. For example, the inclusion of NUMT variants in a population study may cause an incorrect connection between the NUMT and population variation. On the other hand, a missed PHP could cause an incorrect diagnosis or prognosis of a disease, especially when the severity of the disease is influenced by the level of heteroplasmy known as the threshold effect [54]. Similarly, undetected authentic heteroplasmies could create differences between an unknown sample and a reference sample in a forensic case, potentially resulting in a false exclusion [55].

## 4. Materials and Methods

### 4.1. Samples and Whole-Genome Sequencing

The WGS data for the 942 SweGen individuals [30] from the TwinGene project within the Swedish Twin Registry [31] were analyzed to generate mitogenome haplotypes. The WGS data were generated from blood samples as described in Ameur et al. [30]. Briefly, DNA was fragmented using a Covaris E220 sonicator (Covaris Inc., Woburn, MA, USA), followed by automated library preparation using the TruSeq DNA PCR-Free Library Preparation Kit (Illumina, San Diego, CA, USA). Paired-end sequencing (150x150) was performed on an Illumina HiSeq X instrument with v2.5 sequencing chemistry. Reads were aligned using BWA-MEM 0.7.12 [56] to the GRCh37 version of the human reference genome, which included chrMT (mitochondrial reference genome, or rCRS). Alignments for the same sample from different sequencing events were merged and then processed with version 3.3 of the GATK software suite [57], including duplicate removal, to produce a final WGS BAM file for each sample.

### 4.2. Mitochondrial Genome Haplotype Generation

Sequences that mapped to chrMT of GRCh37 in the WGS BAM files were imported into CLC Genomics Workbench v12.0.1 (QIAGEN, Hilden, Germany). The consensus mapping approach that was employed is similar to the workflow described in Ring et al. [23], which was designed to minimize the inclusion of reads dissimilar to the sample haplotype (i.e., NUMTs). The mtDNA reads from the WGS BAM were mapped to a circularized-version of the rCRS using default mapping parameters (0.5 length fraction, 0.8 similarity fraction). Based on this mapping, a consensus (major) sequence was generated for each sample. All the WGS mtDNA reads for each sample were then mapped to their respective consensus sequence using highly stringent parameters (0.95 length fraction, 0.95 similarity fraction). Consensus-mapped reads were then realigned to the rCRS for variant calling. Variant detection was performed using the Low Frequency Variant Detection tool, employing a minimum read depth of 100X with quality filtering (neighborhood radius of 5 bp, minimum central/neighborhood quality of 30) and 5% minor nucleotide frequency for heteroplasmy detection. These minimum thresholds were utilized in this study as they were shown to produce reliable mitogenome data in Taylor et al. [8]. The 100X read depth threshold is higher than most WGS (and capture) studies that use low coverage data (e.g., 10X, 30X). However, low coverage data may be prone to other artifacts that could complicate the assessment of NUMTs, especially when using a 5% frequency threshold. Furthermore, an initial assessment of the data noted that more than 90% of the SweGen samples had minimum read depths 100X or greater coverage.

The AFDIL-QIAGEN mtDNA Expert, or AQME, (v2.1.1) tools [58] were utilized to shift indels 3′ within homopolymer or repeat regions and convert the variant track into a mitogenome haplotype according to forensic nomenclature [53]. Variants were reported based on differences from the rCRS (e.g., A263G, where A is the rCRS nucleotide and G is the variant) and mixed positions were automatically denoted with IUPAC nucleotide codes. Additional modifications included filtering of homopolymer regions to automatically report the major molecule when LHP was observed [53]. To further assist with LHP regions, the AQME read count analysis tool and a custom Excel (Microsoft Corporation, Redman, WA, USA) template was utilized to confirm the major length molecule and appropriate indel nomenclature in homopolymer regions [8,59]. Low-level heteroplasmy (<10%) was consistently observed in the polyadenine (A-) stretches preceding the C-stretches of the HVS1 and HVS2 regions. For example, A302M was observed in nearly all samples, as was A16183M in samples with a T16189C variant. These heteroplasmic variants are likely artifacts caused by post-homopolymer errors observed in HiSeq X sequencing [60]; therefore, heteroplasmies in these A-stretch regions preceding the HVS C-stretches were not included in the final mtDNA haplotype.

Indel alignment issues in C-stretches, specifically HVS1 and HVS2, resulted in read depths below threshold (100X) at several positions in these regions. If fewer than five positions were below the read depth threshold, then the alignment and read count analysis output were inspected to ensure sufficient high-quality reads across the entire LHP region. Based on the review of the one to four positions below the read depth threshold, the nucleotide at these added positions was confirmed and the entire mitogenome was reported (nps 1–16569). Additionally, the AQME Mitochondrial Haplogrouper tool was used to predict the mtDNA haplogroup based on Phylotree Build 17 [34,61].

### 4.3. Classification of Mixed Positions

Multiple assessments were performed to identify NUMTs in the mitogenome haplotypes produced from WGS data. The first (initial) review of mixed (i.e., heteroplasmic) positions detected (≥5%) in the mitogenome haplotypes was performed in CLC during the independent review of the data by two experienced analysts. They assessed variant metrics such as base quality and forward/reverse balance of all mixed positions. Heteroplasmies detectable with the 2% frequency threshold were also reviewed by the analysts to evaluate the presence of NUMT reads and other artifacts below the 5% minimum frequency threshold but above background noise. Furthermore, mixed positions were inspected in the read mappings to assess the presence of in-phase/associated variants and indel alignment issues. Mixed positions that could not be classified as NUMT variants were maintained in the mitogenome haplotype for further review.

Following the initial review of the mitogenome haplotypes from the SweGen WGS data, assessments continued to focus on variants that could possibly be associated with NUMT reads. To enable these assessments, all mixed positions were compiled with detailed information such as the position, minor nucleotide, and minor nucleotide frequency. Minor variants were reported based on differences from the rCRS (e.g., A263G). If the minor nucleotide was consistent with the rCRS, it was reported with the rCRS nucleotide and position (e.g., A263). All mixed positions remaining in the mtDNA haplotypes after the initial review were critically assessed based on these five criteria: (1) minor nucleotide frequency, (2) NUMT variants documented in the study by Li et al. [25], (3) NUMT variants identified during the initial review of the SweGen data, (4) occurrence (≥5%) in other haplotypes, and (5) observations in the low frequency SweGen data (2–5%). The known NUMT variants used for this assessment were based on the more 10,000 variants associated with NUMTs in the Li et al. study [25]. Opposed to other studies that provide a collection of known NUMT sequences (e.g., [2,3]), the list of NUMT variants included as supplementary material by Li et al. provided an easily implemented NUMT variant catalog for comparison. Sample-specific characteristics were also considered during the assessment of the remaining mixed positions, such as the total number of PHPs included in the mtDNA haplotype, whether any NUMT variants were identified during the initial review (≥5%) or detectable (≥2%) in the sample, and the average read depth. Overall, a holistic approach was employed in the evaluation of the five mixed position assessment criteria and sample-specific characteristics. Mixed positions that could not be associated with NUMTs were ultimately classified as probable PHPs and maintained in the mtDNA haplotype.

### 4.4. Additional Quality Control Measures

Pairwise comparisons were performed to identify shared haplotypes (ignoring indels and heteroplasmy). If samples with shared haplotypes were determined to be closely related (i.e., first- or second-degree relatives) based on kinship coefficients calculated from the nuclear data [30], then only one mitogenome haplotype from related individuals was included in the dataset. After these QC steps, the dataset was submitted to the European DNA Profiling Group’s mtDNA Population Database (EMPOP) for additional QC checks and confirmation of the AQME haplogroup assignments [43,62]. The SweGen samples were ultimately included in this study dataset, and considered “passing”, based on the review of the mtDNA data and assessment of the following QC criteria: complete or nearly complete (≤4 positions below threshold) coverage of the entire mitogenome at 100X, single source (e.g., minimal number of PHPs, high major nucleotide frequencies at most variant positions), and maternally unrelated to other passing samples in the dataset. Samples that did not meet these QC criteria were excluded from further analysis.

Since haplotype confirmation with a NUMT-minimizing method (e.g., long-range amplification) was not possible for the SweGen samples, the mitogenome data using both the 5% and 10% frequency thresholds were compared to two previous high-quality mitogenome datasets. The “platinum-quality” NGS data of over 1300 U.S. mitogenome haplotypes presented in Taylor et al. [8] was the first dataset, which was analyzed with a 5% frequency threshold and involved long-range amplification for mitogenome enrichment. The second dataset (Just et al. [9]) includes 588 U.S. mitogenomes produced with an 8-amplicon enrichment strategy [45] and Sanger sequencing, permitting a heteroplasmy detection of approximately 10% due to the qualitative analysis of Sanger data. A 10% frequency threshold was also applied to the Taylor et al. dataset to allow for direct comparison to the other data (i.e., Just et al. Sanger data and the 10% SweGen data). Additionally, the datasets were compared to the blood and skin specimen data from Li et al. [11]. These NGS data were generated using capture enrichment of the mitogenome and high-throughput sequencing on an Illumina HiSeq2000 platform. The Li et al. study utilized a 0.5% frequency threshold [11], but 5% and 10% thresholds were applied to the Li et al. haplotype data for comparison to the other datasets.

### 4.5. Data Analysis

Outputs from the CLC Genomics Workbench were exported to Excel (Microsoft, Redmond, WA, USA) and various analysis metrics were calculated, including average variant frequency and average read depth. The metrics and other details were stored in Access (Microsoft), which was utilized to determine summary metrics. The distribution of mixed positions classified as NUMT variants and PHPs was visualized across the mitogenome using the circlize package v0.4.10 in R version 4.0.2 software (Foundation for Statistical Computing, Vienna, Austria) [63,64].

## 5. Conclusions

Whole-genome sequencing data are a largely underutilized resource of mitogenome haplotypes useful for genetic research, including population genetics, medical studies and forensic applications. However, the generation of accurate haplotypes from the SweGen WGS data proved difficult at a 5% frequency threshold, even with specialized bioinformatic workflows and multiple assessments to prevent the inclusion of low-level variants caused by NUMTs. An elevated frequency threshold of 10% would eliminate >96% of the NUMT interference observed with the 5% frequency threshold. The 13 NUMT variants that exceeded the 10% threshold were localized to a NUMT hotspot region and were clearly distinguishable from PHPs. Furthermore, no heteroplasmic variants above 10% were classified as a NUMT variant in one sample and a PHP in another, making the mtDNA analysis of the WGS data even more straightforward. As a result, lower coverage data (such as the incomplete haplotypes excluded for this study) may be suitable for the generation of high-quality mitogenome data. Approximately 45% of the PHPs were not detected with the 10% frequency threshold. Though there would be a loss in heteroplasmic variation with the application of the elevated threshold, the resulting haplotypes were consistent with high-quality mitogenome datasets. Therefore, it is possible that at least a portion of the PHPs included in the haplotypes generated using a 5% threshold were actually due to NUMTs. Based on these findings, the use of a 10% minimum frequency threshold for mtDNA analysis of the SweGen WGS data was necessary to produce reliable mitogenome haplotypes. However, future tools that can effectively eradicate NUMT reads or prune NUMT variants may allow for the application of lower detection thresholds as well as streamline the mtDNA analysis of WGS data.

## Figures and Tables

**Table 1:** Average analysis metrics for each sample category based on the mitochondrial DNA (mtDNA) analysis of the SweGen dataset. The passing category includes complete (all positions with at least 100X read depth) and nearly complete (one to four positions below 100X) haplotypes that met all quality control criteria. Incomplete haplotypes (five or more positions below 100X), possible mixtures and related samples were excluded from the analysis. The “% Mapped Reads” is the proportion of total reads that mapped to the GRCh37 and the “% mtDNA Reads” is the proportion of the mapped reads that aligned to the mtDNA reference genome.

Category	Subclassification	Count	Total Reads	% Mapped Reads	% mtDNA Reads	Average Read Depth
Passing	Complete	858	865,518,368.5	99.16%	0.036%	2365.9
	Nearly Complete	59	833,866,169.5	99.20%	0.010%	652.5
Excluded	Incomplete	17	784,241,829.7	99.32%	0.010%	592.0
	Mixed	7	762,731,157.9	98.72%	0.019%	1191.1
	Related	1	869,537,450	99.52%	0.029%	1870.7

**Table 2:** Average analysis metrics of passing samples depending on the observation of NUMT variants in the mtDNA data. The “% Mapped Reads” is the proportion of total reads that mapped to the GRCh37 and the “% mtDNA Reads” is the proportion of the mapped reads that aligned to the mitochondrial reference genome.

NUMT Variants	Count	Total Reads	% Reads Mapped	% Mapped mtDNA	Average Read Depth
None	561	869,724,465.0	99.17%	0.044%	2919.1
2–5%	265	854,695,905.9	99.16%	0.021%	1390.1
≥5%	91	855,009,718.4	99.16%	0.011%	686.1

## Data Availability

The SweGen mitogenome haplogroups are presented in Table S1 and mitogenome haplotype data are available upon request.

## Supplementary Materials

The following are available online at https://www.mdpi.com/article/10.3390/ijms23042244/s1.
